# Associations of Infant Feeding with Body Composition and Cardiometabolic Health in Young Female University Students

**DOI:** 10.1089/jwh.2021.0464

**Published:** 2022-09-15

**Authors:** Mari Honda, Ayaka Tsuboi, Satomi Minato-Inokawa, Mika Takeuchi, Miki Kurata, Tomofumi Takayoshi, Yushi Hirota, Bin Wu, Tsutomu Kazumi, Keisuke Fukuo

**Affiliations:** ^1^ Open Research Center for Studying of Lifestyle-Related Diseases, Mukogawa Women's University, Nishinomiya, Japan.; ^2^ Department of Health, Sports, and Nutrition, Faculty of Health and Welfare, Kobe Women's University, Kobe, Japan.; ^3^ Research Institute for Nutrition Sciences, Mukogawa Women's University, Nishinomiya, Japan.; ^4^ Department of Nutrition, Osaka City Juso Hospital, Osaka, Japan.; ^5^ Center for the Promotion of Interdisciplinary Education and Research, Kyoto University, Kyoto, Japan.; ^6^ Department of Food Sciences and Nutrition, School of Food Sciences and Nutrition, Mukogawa Women's University, Nishinomiya, Japan.; ^7^ Division of Diabetes and Endocrinology, Kobe University Graduate School of Medicine, Kobe, Japan.; ^8^ Department of Endocrinology, First Affiliated Hospital of Kunming Medical University, Kunming, China.; ^9^ Department of Medicine, Kohnan Kakogawa Hospital, Kakogawa, Japan.

**Keywords:** formula feeding, LDL cholesterol, resting heart rates, female university students, adiponectin

## Abstract

**Background:**

We assessed the association of infant feeding with body composition and cardiometabolic health at 20 years in a setting where infant feeding is not associated with socioeconomic status.

**Materials and methods:**

Body size trajectory since birth, current body composition measured using whole-body dual-energy X-ray absorptiometry, and a broad range of cardiometabolic risk factors were compared cross-sectionally among young female university students who were ever breastfed (*n* = 158, 120 exclusively, and 38 mainly), mixed fed (*n* = 124), and formula fed (*n* = 15, 10 mainly, and 5 exclusively)

**Results:**

Compared with breastfed and mixed fed women, formula fed women had higher serum total and low-density lipoprotein (LDL) cholesterol although fat mass, fat distribution, fasting glucose, and insulin and high-density lipoprotein cholesterol did not differ. In addition, resting heart rates were higher in formula fed women compared with the other two groups of women although systolic and diastolic blood pressure did not differ. Further, formula fed women had higher adiponectin while serum leptin did not differ. There was no difference in birthweight, weight and height in childhood and adolescence, and glucose tolerance. On multivariate logistic regression analysis, formula feeding was associated with resting heart rates (odds ratio [OR]: 1.06, confidence interval [95% CI]; 1.01–1.12, *p* = 0.01) and adiponectin (OR: 1.3, 95% CI; 1.1–1.5, *p* < 0.001) independently of serum total and LDL cholesterol.

**Conclusions:**

Breastfeeding may be associated with favorable lipid profile and autonomic nervous function in young adults through mechanisms unrelated to adiposity, implicating potential long-term benefits of breastfeeding for cardiovascular health. Higher adiponectin in nonbreastfed women warrants further studies.

## Introduction

Low resting heart rate is associated with lower risk for cardiovascular and noncardiovascular premature mortality.^1,2^ Although the resting heart rate is a highly variable parameter that is influenced by numerous factors, including sex (higher in women), obesity, waist girth, and blood pressure (BP),^3^ the resting heart rate is mainly determined by cardiac vagal control.^4,5^ It is well known that endurance training is associated with low resting heart rate.^6^ Breastfeeding shifts maternal and infant's autonomic nervous system balance toward relatively greater parasympathetic and lesser sympathetic activity; the opposite occurs with bottle feeding.^7,8^ It has been reported that repeated vagal tone measurements (assessed via heart rate recovery) were associated with breastfeeding and physical activity at preschool age.^9^ However, there is a dearth of evidence in adults due probably to many confounders.

Breast milk contains more cholesterol than infant formula,^10^ and breastfed infants had increased plasma concentrations of total cholesterol, low-density lipoprotein (LDL) cholesterol, and reduced endogenous cholesterol synthesis.^11,12^ Long-term consequences of breastfeeding on cholesterol were inconclusive: initial breastfeeding (particularly when exclusive) was associated with lower blood cholesterol in some^13^ but not in other systematic reviews.^14,15^ These were based largely on observational studies, which were carried out in mostly high-income countries from western world, and studies from developed Asian countries are limited.^16,17^ Because some studies suggested a stronger effect in women,^9,17,18^ the present study tested whether infant feeding was associated with body composition and cardiometabolic health, including lipid profile and a marker of autonomic nervous function in young Japanese female university students of Mukogawa Women's University, in whom the socioeconomic status was less heterogenous.^19^

For example, >90% of grade 1 students are 18 years old. This approach may decrease the interference of age and environmental factors, including smoking, alcohol, educational, and socioeconomic status. Further, in almost all students, almost all school expenses were covered by parents, suggesting that socioeconomic status appears to be less heterogeneous among mothers who fed participants of the present study. We assessed current dietary intake of saturated fatty acids and cholesterol, which may be related to blood lipid profile.^20^

## Materials and Methods

We studied cross-sectionally 279 female Japanese university students of Mukogawa Women's University among previously reported 481 students.^21^ They participated as a volunteer and provided data on infant feeding during the first year after delivery. Their biological mothers chose one of the following five response options which we provided: exclusively and mainly breastfeeding, mixed feeding (half breastfeeding and half formula feeding), and mainly and exclusively formula feeding. Birthweight, height, and weight at age 12 and 15 years were also provided either through maternal health check notes or child health notebook records (issued by each municipal office). Subjects with clinically diagnosed acute or chronic inflammatory diseases, endocrine, cardiovascular, hepatic, renal diseases, hormonal contraception, and unusual dietary habits were excluded. Nobody reported to receive any medications or have regular supplements.

The study was approved by the Ethics Committees of the Mukogawa Women's University (No. 07-28 on 19/02/2008) to be in accordance with the Declaration of Helsinki. All subjects were recruited as volunteers and gave written consent after the experimental procedure had been explained.

After a 12-hour overnight fast, participants underwent blood sampling, measurement of anthropometric indices, BP, and body composition as previously described.^20–23^ Systolic and diastolic BP and pulse rates were measured using an automated sphygmomanometer (BP-203RV II; Colin, Tokyo, Japan) after participants were seated at least for 5 minutes. The measurements were repeated after 2–3 minutes, and the average of the measurements was used for analyses. Although heart rates were not measured, pulse rates may be equal to heart rates in young healthy women. Therefore, we used heart rates expressed in beats per minute (bpm) instead of pulse rates in the present study. A standard 75 g oral glucose tolerance test (OGTT) was done in 106 women. The blood was withdrawn at 0 (fasting), 30 minutes, 1 hour, and 2 hours for glucose and insulin measurements.

The area under the concentration curve of glucose (AUCg) and insulin (AUCi) was calculated with the trapezoidal method. Serum total and high-density lipoprotein (HDL) cholesterol, triglycerides, adiponectin, leptin, and high-sensitivity C-reactive protein (hsCRP) were assayed in fasted samples.^21^ LDL cholesterol was calculated by the Friedewald's formula. Plasma glucose was determined by the hexokinase/glucose-6-phosphate dehydrogenase method (interassay coefficient of variation [CV] <2%). Serum insulin was measured by an ELISA method with a narrow specificity excluding des-31, des-32, and intact proinsulin (interassay CV <6%). Markers of insulin resistance included fasting insulin and homeostasis model assessment-insulin resistance (HOMA-IR).^24^

Fat mass, bone mass, and lean mass for arms, legs, trunk, and the total body were measured using whole-body dual-energy X-ray absorptiometry (DXA) (Hologic QDR-2000, software version 7.20D, Hologic, Inc. Waltham, MA) as previously reported.^21^ The leg region included the entire hip, thigh, and legs. General adiposity was assessed by fat mass index calculated as body fat mass in kg divided by height in meters squared. Abdominal fat accumulation was assessed by trunk/leg fat ratio.^25^ Because lean mass in arms and legs represents skeletal muscle mass, a sum of the two was used as appendicular skeletal muscle mass (ASM). Height-adjusted skeletal muscle mass was also assessed by skeletal muscle mass index, calculated as ASM in kilograms divided by squared height in meters.

Dietary intake of the previous month was assessed using the self-administered diet history questionnaire.^26^ This has been widely used throughout Japan and its validity with respect to commonly studied nutrition factors has been confirmed.

The data are presented as mean ± SD. Due to deviation from normal distribution, fasting insulin, HOMA-IR, and hsCRP were logarithmically transformed for analyses. Because women with mainly and exclusively formula feeding were small in number (*n* = 10 and 5, respectively), the two groups were combined for analyses (formula fed). As number of women mainly breastfed (*n* = 38) were small, exclusively (*n* = 120) and mainly breastfed women were combined for analyses (breast fed). Differences among three groups were analyzed using analysis of variance and then Bonferroni's multiple comparison procedure. Multivariate logistic regression analyses were done for formula feeding as a dependent variable. The independent variables included were those that displayed significant differences between breastfeeding and formula feeding.

A two-tailed value of *p* < 0.05 was considered significant. The statistics were performed with SPSS system 21 (SPSS, Inc., Chicago, IL), except for *post hoc* power analyses, which were done using system 28.

## Results

As previously reported^20–23^ and shown in Tables 1–3, young women were of normal weight, normoglycemic, normolipidemic, and normotensive. The distribution of women who were breastfed (*n* = 158, 53%), mix fed (*n* = 124, 42%), and formula fed (*n* = 15, 5%) were similar to the distribution reported in the national nutrition survey for babies and infants in 2015 by the Ministry of Health, Labor, and Welfare, Japan (56%, 34% and 10%, respectively).

**Table 1.** Weight Trajectory and Body Composition at 20 Years in Breastfed, Mixed-Fed, and Formula-Fed Japanese Female University Students

	Breastfed	Mixed fed	Formula fed	*p*
	*n* = 158	*n* = 124	*n* = 15	
Age (years)	19.9 ± 1.2	19.9 ± 1.2	20.3 ± 1.0	0.517
Birth weight (g)	3257 ± 427	3224 ± 427	3071 ± 313	0.252
Weight (kg) at age 12	44.7 ± 7.9	45.2 ± 7.5	49.3 ± 7.3	0.132
At age 15	51.8 ± 6.8	51.9 ± 7.5	55.0 ± 7.1	0.292
At age 20	55.0 ± 7.4	54.9 ± 7.3	56.3 ± 9.5	0.775
Height (cm) at age 12	153 ± 8	154 ± 7	156 ± 7	0.202
At age 15	160 ± 6	160 ± 6	161 ± 8	0.716
At age 20	162 ± 6	161 ± 6	162 ± 8	0.624
Body mass index (kg/m^2^) at age 12	19.0 ± 2.4	19.1 ± 2.2	20.1 ± 1.8	0.305
At age 15	20.2 ± 2.1	20.3 ± 2.1	21.1 ± 1.5	0.296
At age 20	20.9 ± 2.1	21.0 ± 2.0	21.3 ± 2.5	0.828
Body fat percentage (%)	25.4 ± 5.9	25.7 ± 6.1	26.3 ± 7.0	0.796
Fat mass index (kg/m^2^)	5.3 ± 1.6	5.4 ± 1.6	5.6 ± 1.9	0.808
Trunk/leg fat ratio	1.2 ± 0.2	1.2 ± 0.3	1.2 ± 0.2	0.581
ASMI (kg/m^2^)	6.4 ± 0.9	6.4 ± 0.8	6.4 ± 1.0	0.916
Athletes, *n* (%)	73 (46.2)	63 (50.8)	5 (33.3)	0.395

The values are represented by mean ± SD.

ASMI, appendicular skeletal muscle mass index.

**Table 2.** Lipid Profile, Blood Pressure, Resting Heart Rate, and Serum Adipokines in Breastfed, Mixed-Fed, and Formula-Fed Japanese Female University Students

	Breastfed	Mixed fed	Formula fed	*p*
	*n* = 158	*n* = 124	*n* = 15	
Total cholesterol (mg/dL)	181 ± 30	180 ± 22	200 ± 31	0.032
LDL cholesterol (mg/dL)	93 ± 23	93 ± 20	111 ± 29	0.011
HDL cholesterol (mg/dL)	76 ± 13	77 ± 14	78 ± 10	0.857
Triglycerides (mg/dL)	58 ± 24	54 ± 20	52 ± 17	0.381
Systolic BP (mmHg)	107 ± 11	104 ± 8	103 ± 9	0.067
Diastolic BP (mmHg)	60 ± 8	58 ± 6	64 ± 5	0.009
Resting heart rate (bpm)	63 ± 11	60 ± 9	70 ± 8	<0.001
Leptin (ng/mL)	7.8 ± 4.0	7.5 ± 3.3	8.1 ± 4.5	0.727
Adiponectin (mg/L)	11.2 ± 3.9	11.2 ± 4.4	15.4 ± 3.4	<0.001
PAI-1 (ng/mL)	20 ± 11	20 ± 12	24 ± 10	0.354
hsCRP (μg/dL)	29 ± 64	28 ± 74	45 ± 126	0.681
TNF-α (pg/mL)	0.6 ± 0.4	0.6 ± 0.6	0.7 ± 0.3	0.239
Hemoglobin (g/dL)	13.1 ± 0.9	13.1 ± 1.0	13.1 ± 1.0	0.988

The values are represented by mean ± SD.

BP, blood pressure; HDL, high-density lipoprotein; hsCRP, high-sensitivity C-reactive protein; LDL, low-density lipoprotein; PAI-1, plasminogen activator inhibitor-1; TNF-α, tumor necrosis factor-α.

**Table 3.** Glucose and Insulin Responses During a 75 g Oral Glucose Tolerance Test in Breastfed, Mixed-Fed, and Formula-Fed Japanese Female University Students

	Breastfed	Mixed fed	Formula fed	*p*
	*n* = 158 (58)	*n* = 124 (33)	*n* = 15 (15)	
Fasting glucose (mg/dL)	86 ± 6	85 ± 7	85 ± 8	0.435
30 minutes glucose (mg/dL)	122 ± 23	123 ± 32	117 ± 25	0.775
1-hour glucose (mg/dL)	94 ± 28	110 ± 32	96 ± 26	0.054
2 hours glucose (mg/dL)	89 ± 17	90 ± 16	89 ± 18	0.926
Fasting insulin (μU/mL)	6 ± 4	6 ± 5	7 ± 3	0.824
30 minutes insulin (μU/mL)	70 ± 36	45 ± 30	55 ± 29	0.004
1-hour insulin (μU/mL)	46 ± 29	40 ± 25	47 ± 22	0.495
2 hours insulin (μU/mL)	40 ± 26	36 ± 21	43 ± 37	0.635
AUCg (mg/dL/2 hours)	198 ± 37	210 ± 42	196 ± 36	0.289
AUCi (μU/mL/2 hours)	91 ± 39	71 ± 36	86 ± 38	0.060
HbA1c (%)	5.2 ± 0.2	5.2 ± 0.3	5.2 ± 0.2	0.888
HOMA-IR	1.3 ± 0.8	1.3 ± 1.2	1.5 ± 0.9	0.732

The values are represented by mean ± SD.

AUCg and AUCi, area under the glucose and insulin concentration curve, respectively; HOMA-IR, homeostasis model assessment-insulin resistance; Number in parentheses, No. of OGTT; OGTT, oral glucose tolerance test.

Birth weight, weight and height, and hence body mass index at age 12 and 15 did not differ significantly among the three groups of women (Table 1). Although mean values of body weight at age 12 and 15 were higher in formula fed compared with breastfed and mix fed women, these differences were not significant. *Post hoc* power analyses for weight comparison at age 12 and 15 yielded 0.410 and 0.267, respectively, in the setting of the present study, indicating a weak statistical power. Fat mass, fat distribution, muscle mass, and the percentage of athletes did not differ as well.

**FIG. 1.**
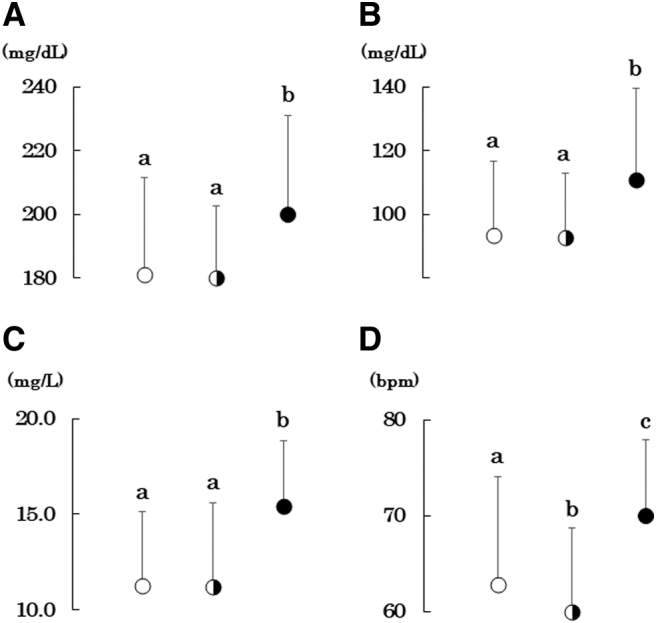
Serum total **(A)** and LDL cholesterol **(B)**, adiponectin **(C)**, and resting heart rate **(D)** in young female university students who were ever breastfed (*n* = 158, *open circles*), mixed fed (*n* = 124, *semiclosed circles*), and formula fed (*n* = 15, *closed circles*). Mean ± SD. Means not sharing a common letter are significantly different with each other at *p* < 0.05 or less by Bonferroni's multiple comparison procedures. LDL, low-density lipoprotein.

Formula fed women had higher serum total and LDL cholesterol compared with breastfed and mix fed women (Table 2 and Fig. 1) although HDL cholesterol did not differ. Formula fed women had higher resting heart rate as well although systolic BP did not differ. Diastolic BP was higher in formula-fed women. However, the difference was significant between formula-fed and mix-fed women (*p* = 0.01). Although serum leptin and body fat percentage did not differ, adiponectin was higher in formula-fed women (Fig. 1). Mean adiponectin concentrations in breastfed and mix-fed women were similar to previously reported mean values in 174 female collegiate athletes and 311 nonathletes (11.6 ± 4.3 and 11.5 ± 4.3 mg/L, respectively).^23^ There was no difference in markers of inflammation. Although total and LDL cholesterol, resting heart rate, and diastolic BP were higher in formula-fed women, they were still within a normal range.

Glucose tolerance, fasting insulin, and HOMA-IR did not differ among the three groups of women although 30 minutes insulin was higher in breastfed compared with mix-fed women (Table 3). There was also no difference in dietary intake of cholesterol and saturated fatty acids (Table 4).

**Table 4.** Dietary Intake in Breastfed, Mixed-Fed, and Formula-Fed Japanese Female University Students

	Breastfed	Mixed fed	Formula fed	*p*
	*n* = 58	*n* = 33	*n* = 15	
Energy (kcal/day)	2311 ± 995	2103 ± 708	1913 ± 437	0.220
Cholesterol (mg/day)	370 ± 220	326 ± 184	334 ± 118	0.562
Carbohydrate (%E)	55.7 ± 8.3	56.5 ± 8.3	53.8 ± 5.7	0.548
Protein (%E)	12.4 ± 2.2	12.0 ± 1.9	13.4 ± 1.9	0.126
Fat (%E)	30.3 ± 6.9	29.5 ± 7.8	31.1 ± 4.7	0.746
SFA (%E)	8.9 ± 2.3	8.3 ± 2.1	9.0 ± 1.9	0.427
MUFA (%E)	10.7 ± 3.0	10.2 ± 3.5	10.9 ± 2.1	0.694
PUFA (%E)	6.3 ± 1.9	6.2 ± 2.7	6.4 ± 1.5	0.968
Fish (g/day)	50 ± 38	45 ± 40	63 ± 31	0.321
Meat (g/day)	78 ± 52	62 ± 37	72 ± 30	0.253
Egg (g/day)	41 ± 41	38 ± 32	37 ± 24	0.900
Dairy (g/day)	153 ± 166	120 ± 111	117 ± 70	0.474

Means ± SD.

%E, % of energy; MUFA, monounsaturated fatty acid, PUFA, polyunsaturated fatty acid; SFA, saturated fatty acid.

Multivariate logistic regression analysis revealed that formula feeding was associated with resting heart rates and adiponectin independently of serum total and LDL cholesterol (Table 5).

**Table 5.** Multivariate Logistic Regression Analysis for Formula Feeding

	OR	95% CI	*p*
		lower–Upper	
Total cholesterol	0.97	0.93–1.02	0.26
LDL cholesterol	1.05	0.99–1.12	0.09
Adiponectin	1.28	1.12–1.47	<0.001
Resting heart rate	1.07	1.01–1.12	0.01

CI, confidential interval; OR, odds ratio.

## Discussion

The present study has shown that formula feeding was associated with higher total and LDL cholesterol. Further, it was associated with higher serum adiponectin and resting heart rate. Among these variables, serum adiponectin and resting heart rate were associated independently with formula feeding. However, there was no association with weight trajectories since birth, current body fat mass and distribution, dietary intake of saturated fatty acids and cholesterol, glucose tolerance, and inflammatory markers. These findings were observed in Japanese female students of Mukogawa Women's University, where >95% of grade 1 students are 18 years old.^20,21^ This approach may decrease the interference of age and environmental factors, including smoking, alcohol, educational, and socioeconomic status.

The mechanism underlying the relationship between formula feeding and higher serum adiponectin is unknown. It has been reported that longer exclusive breastfeeding has a beneficial effect on cardiorespiratory fitness in children and adolescents.^27,28^ Paradoxically, serum adiponectin levels are inversely associated with cardiorespiratory fitness in nonoverweight, but not in overweight adolescents.^29,30^ These observations may be related to our finding of higher adiponectin in formula-fed young normal weight women, although we did not measure cardiorespiratory fitness.

As previously summarized,^8^ the vagus nerve, a component of the parasympathetic nervous system, innervates musculature related to sucking, swallowing, and breathing. Breastfeeding, relative to bottle-feeding, requires more effortful and frequent sucking, which may contribute to the development of higher resting vagal tone. It has been reported that vagal tone measurements (assessed via heart rate recovery) were associated with breastfeeding and physical activity at preschool age.^9^ In the present study, resting heart rates, a crude index of the autonomic nervous system tone, reflecting a balance of sympathetic and parasympathetic inputs on the heart, were lower in breastfed and mixed fed compared with formula-fed young female university students, suggesting relatively higher vagal tone in the former two groups of women, although we did not measure autonomic nerve function.

Long-term consequences of breastfeeding on blood cholesterol were inconclusive in studies which were carried out in mostly high-income countries from western world^13–15^ and studies from developed Asian countries are limited where the prevalence of overweight and obesity was lower compared with western countries. We found two studies from developed Asian countries.^16,17^ Consistent with our observation, Japanese middle-aged women fed formula milk, but not men, experienced higher LDL cholesterol levels than women fed breast milk or a mixture of breast and formula milk.^17^ Hui et al. reported that exclusive breastfeeding, but not mixed feeding at 0 to 3 months, compared with formula feeding was associated with lower total and LDL cholesterol at 17.5 years in 3261 participants in the Hong Kong Chinese birth cohort Children of 1997.^16^

As reviewed by Owen et al.,^13^ breastfeeding is associated with increased total and LDL cholesterol in infancy, but lower levels in adulthood/adult life, suggesting nutritional programming of cholesterol synthesis by exposure to breast milk in early life, although the biological mechanism for this process remains unclear.

The strengths of the present study include a homogeneous study population with scarce confounding factors as previously reported^20,21^ and accurate and reliable measures of DXA-derived body composition. Several limitations of this study warrant consideration. The number of participants, specifically formula-fed women, was small, whereas a broad range of confounders were adjusted, including weight trajectories since birth, current body fat mass and distribution, dietary intake, and socioeconomic status. The cross-sectional design of the present study complicates the drawing of causal inferences, and a single measurement of biochemical variables may be susceptible to short-term variation, which would bias the results toward the null. The recruitment procedure may also have some potential impact on the results.

As the participation was voluntary, women who pay more attention to health may be more likely to participate. We used crude measures of insulin sensitivity/insulin resistance, which may be less accurate. Statistical power was not calculated. As we studied young Japanese women only, results may not be generalized to other genders, age populations, races, or ethnicities.

## Conclusions

Breastfeeding in infancy may be associated with favorable lipid profile and autonomic nervous function in young adults through mechanisms unrelated to adiposity, implicating potential long-term benefits of breastfeeding for cardiovascular health. Higher adiponectin in nonbreastfed women warrants further studies.
